# An acute multidisciplinary treatment strategy for patients with a hip fracture

**DOI:** 10.1186/1757-7241-20-S2-P5

**Published:** 2012-04-16

**Authors:** Claus Rosenby Mortensen

**Affiliations:** 1 Akutafdelingen, Nykøbing F. Hospital, Sygehus Syd, Region Sjælland, Denmark

## Background

The "Reference Program for Patients with Hip fractures" from 2008 recommends a multidisciplinary treatment strategy in patients with hip fractures in terms of follow-up treatment and rehabilitation, but there is no focus on acute treatment of this patient group in the Danish reference program.

The project aims to investigate whether it is possible to implement an acute multidisciplinary treatment strategy that can help reduce the mortality rate among patients with a hip fracture in an emergency department.

## Methods

Intervention study with retrospective control group. Process indicators are compiled by auditing techniques, as a qvasi-experimental design.

Inclusion criteria: All patients > 65 years are received with suspected hip fracture caused by direct trauma to the hip/pelvic region, which has shortening and external rotation of the leg.

Exclusion Criteria: Patients who cannot give consent.

Intervention:

- Fluid therapy started within 15 minutes after arrival.

- Oxygen Therapy.

- Fascia iliaca compartment block.

- X-rays taken within 60 minutes.

- Placement of pressure-relieving mattress.

Data collection: Study Checklist, measurement of vital parameters, patient journal.

## Results

All patients were triaged orange and were followed by measuring vital parameters. 16 weeks after the intervention, the study shows that 46 patients (10 men {68-96}, 36 women {69-96}), 91% have gotten peripheral vein catheter, 72 % have been infused fluid and 67 % had nasal oxygen. 54 % was VAS- scored, and 41% of patients have gotten fascia iliaca compartment block. 25% of the patients underwent X-rays within the 60 minutes (average 1 hour and 30 minutes, range 42 minutes-1 hour and 42 min).

Process indicators are displayed over time, increasing deployment. 88% is put on the pressure-relieving mattress. Background mortality was an index of 1.49 in 2009. After the first 12 weeks of intervention none were dead.

Bias: It has not been possible to obtain data on 11 patients.

## Conclusion

Implementation of the interventions requires continued focus on supervision and training of staff to complete fulfilment. Experience shows that the checklist is not always filled, but that data can be found in the patient record. It is too early to comment on whether patient mortality is reduced.

